# Intensive care unit-acquired dysphagia – change in feeding route after a standardized dysphagia assessment in neurocritical care patients

**DOI:** 10.1038/s41598-024-81529-1

**Published:** 2024-12-02

**Authors:** Sarah Christina Reitz, Joanna Marly, Vanessa Neef, Jürgen Konczalla, Marcus Czabanka, Christian Grefkes-Hermann, Christian Foerch, Sriramya Lapa

**Affiliations:** 1 Department of Neurology, Goethe University Frankfurt, University Hospital Frankfurt, Frankfurt/Main, Germany; 2 Department of Neurosurgery, Goethe University Frankfurt, University Hospital Frankfurt, Schleusenweg 2-16, 60528 Frankfurt/Main, Germany; 3 Department of Anaesthesiology, Intensive Care Medicine and Pain Therapy, Goethe University Frankfurt, University Hospital Frankfurt, Frankfurt/Main, Germany; 4 Clinic of Neurology, Ludwigsburg, Germany

**Keywords:** Feeding route, Dysphagia, Mechanical ventilation, Intubation, Swallowing disorders, Aspiration, Nutrition therapy, Neurology, Risk factors

## Abstract

Background: Dysphagia is a frequent finding on intensive care units (ICUs) and is associated with increased reintubation rates, pneumonia, and prolonged ICU-stay. Only a limited numbers of ICUs have access to a Speech and Language Pathologist (SLP). Hence, it falls upon the critical care team to estimate dysphagia risk and define the safest feeding route. Therefore, the aim of this study was to evaluate if the feeding route established by the ICU-team is changed after a standardized dysphagia assessment (DA) by an SLP. Furthermore, we tried to identify predictors for the need of a SLP assessment looking at the change in feeding route (CIFR) after DA. Methods: We performed a retrospective analysis of patients consecutively admitted for at least 48 h in 2018, to the ICU of the Department of Neurology and Neurosurgery. Following variables were assessed: Referral to an SLP, feeding route before and after DA by an SLP, main diagnosis, and ventilation parameters. Results: From 497 included patients (65 years, IQR 51–77), 148 received a DA, confirming dysphagia in 125 subjects. DA by the SLP resulted in a significant CIFR, with 32 (21.6%) subjects receiving stricter diets, and in 29 (19.6%) cases a reduction of dietary recommendations. 50 patients lacked readiness for oral intake due to severely affected oral phase or reduced consciousness. Conclusion: Dysphagia is a frequent finding in the Neuro-ICU. Assessment of dysphagia-risk and safest feeding route differ significantly between the SLP and the critical care team. Besides implementation of standardized operating procedures for DA, the presence of ICU-specific trained SLP should be mandatory.

**Supplementary Information:**

The online version contains supplementary material available at 10.1038/s41598-024-81529-1.

## Introduction

Dysphagia, including post-extubation dysphagia (PED), is a common condition in hospitalized patients in intensive care units (ICUs). Depending on the study design, patient selection and dysphagia assessment, the reported incidence ranges from 3 to 62% ^1^. In neurological and neurosurgical ICUs (Neuro-ICUs), an even higher dysphagia rate of up to 90% has been described^1^.

Multiple mechanisms, such as trauma caused by endotracheal and tracheostomy tubes, neuromyopathy, reduced laryngeal sensation, altered level of consciousness, gastroesophageal reflux, and dyssynchronous breathing and swallowing have been identified as underlying causes for the development of ICU-dysphagia^3–5^. Critical illness dysphagia is known to be independently associated with adverse patient-centred clinical outcomes, such as (aspiration-) pneumonia, need for reintubation, malnutrition, prolonged ICU and hospital stay, as well as increased healthcare expenditures^6^. Moreover, an excess 90-day all-cause mortality rate of 9.2% is related to the presence of dysphagia^7,8^.

Despite the clinical relevance, ICU-specific guidelines for prevention, assessment, evaluation, and/or treatment of dysphagia in critically ill patients is lacking^9,10^. In most cases, the feeding route is determined by the ICU staff. A recent study by Spronk et al.^9^ evaluated dysphagia in intensive care by an international cross-sectional study, and found that beyond limited awareness of frequency of ICU-dysphagia by the ICU clinicals, the majority of ICUs (67%) had not implemented any routine dysphagia protocol^9,10^. In 66% of the participating ICUs, speech and language pathologist (SLP) consultation were available, but only 4% had access to an assigned SLP. Instrumental swallowing assessment e.g., flexible endoscopic evaluation of swallowing (FEES), which is known to offer high sensitivity in detecting dysphagia and aspiration, was only used in 8% of the survey ICUs (8%)^8–13^.

In summary, a standardized dysphagia protocol has not been implemented in most ICUs. Furthermore, limited access to both a sophisticated dysphagia assessment, by an ICU-specific trained SLP, and an instrumental dysphagia diagnostic could affect the determination of the safest feeding route for ICU patients^9,14^. In most ICUs a multi-professional team (physicians, nurses) is responsible for defining the appropriate feeding route, though it remains unclear on which grounds nutrition recommendations are made^9,15^. Without detailed swallowing evaluation, dysphagic symptoms might be neglected, resulting in inadequate dietary recommendation, placing patients at risk for aspiration and/or malnutrition, impacting patient outcome significantly^1,6,16–18^.

Therefore, the aim of this study was to investigate in how many patients the dysphagia assessment (DA) conducted by an SLP leads to a change in the feeding route (CIFR). Furthermore, we tried to identify clinical predictors for both a CIFR and ICU-dysphagia, in a neuro-intensive care population.

## Methods

### Study design

This study was conducted at the University Hospital Frankfurt, by a certified neurological and neurosurgical intensive care unit (hereinafter referred to Neuro-ICU), with attached trauma centre, Endovascular thrombectomy (EVT)-capable stroke centre and 24/7 thrombectomy, and surgery capacity. The study was conducted according to the principles of the Declaration of Helsinki and with the requirements by the local ethics committee. It was approved by the institutional review board and ethics committee of the Goethe University Hospital Frankfurt (approval no. 2021-80). Written informed consent was waived due the retrospective character of the study by the institutional review board of the Goethe University Hospital Frankfurt.

### Patients

All patients admitted to the ICU of the Department of Neurology and Neurosurgery of the Goethe University Hospital Frankfurt, between January 2018 and December 2018, were retrospectively evaluated for the fulfilment of the following inclusion criteria: (1) stay on the Neuro-ICU for at least 48 h and (2) undergoing a detailed bedside dysphagia examination (hereinafter referred as dysphagia assessment (DA)) by an SLP during their ICU stay. Patients were referred to the SLP, if the ICU-team suspected dysphagia during the water-swallowing test and the patients were in a medically stable condition for an oral intake^19,20^.

Primary diagnosis leading to admission in the Neuro-ICU were grouped into eight categories: (1) ischemic stroke, (2) nontraumatic intracerebral haemorrhage (ICH), (3) non-traumatic subarachnoid haemorrhage (SAH), (4) infectious encephalitis or meningitis, (5) epilepsy, (6) traumatic brain injury, (7) brain tumor, and (8) other (e.g., neuromuscular diseases, spine diseases, but not primarily neurological or neurosurgical diseases). These data were collected for all patients admitted to the ICU, regardless of whether they have been examined by the SLP or not.

Additionally, Charlson comorbidity index (CCI)^21^ and the Body mass index (BMI) were documented. Degree of disability prior admission was assessed using the modified Rankin Scale (mRS)^22^.

**Fig. 1:**
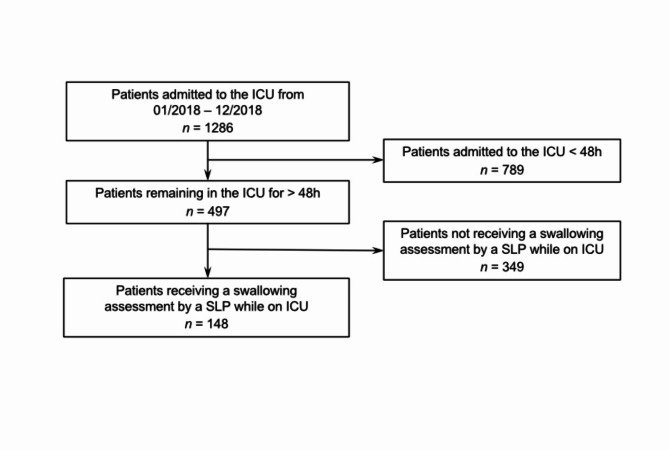
Study flow chart. ICU, intensive care unit; SLP, Speech and Language Pathologist.

Furthermore, reasons for the lack of an SLP consultation were recorded (discharged with mechanical ventilation, palliative procedure/death, unstable medical condition, unknown) as well. All data were obtained from the electronic patient files (admission notes, progress notes, ICU flow sheets, laboratory results, radiologic data, internal diagnostic coding). A study flowchart is provided in Fig. 1.

### ICU parameters

For assessment of disease severity, the Simplified Acute Physiology Score II ^[SAPS II23^ was recorded, as well as total days on invasive mechanical ventilation, and the ventilation mode (endotracheal or via tracheostomy). Furthermore, we collected data on accompanying complications during the ICU stay, such as acute kidney injury, delirium, pneumonia, or any other documented infections.

### Dysphagia assessment by SLP

Dysphagia assessment was performed by trained SLPs, all having > 5 years of experience in ICU care. According to our in-house guidelines, DA in ICU was conducted in a standardized manner as follows.

In a first step, the medical history as well as the current diagnostics results (e.g. blood parameters, radiological findings including chest x-ray, cranial computed tomography, magnetic resonance imaging, course of underlying disease, notes by the treating physician/nurse) were reviewed^24^.

In a second step, the patients´ states of consciousness were evaluated. In those who were alert and responded to voice, the DA was carried out, specifically evaluating the presence of dysarthria, dysphonia, abnormal volitional cough, abnormal gag reflex, as well as the presence of gurgle/wet voice. Furthermore, cranial nerves involved in swallowing (CN V, VII, IX, X, and XII), as well as motor function of the bulbar muscles (e.g., jaw, lips, tongue, hard palate, soft palate, and cheeks), were examined^25,26^.

In a third step, calibrated volumes of water starting with a 5-ml liquid bolus, increasing up to 10-and 20-ml volume were administered to the patient, while assessing the presence of cough, and voice change after swallow. Patients, who did not present with overt airway response, during the single sip water swallowing test (WST), were asked to drink 100 mL of water consecutively, to unmask objective signs of dysphagia, which might have been missed by exclusively applying small volumes^27,28^.

In case of cough or voice change after swallow, dysphagia with signs of aspiration was deemed to be present. In those patients, who were showing sufficient alertness (for at least 15 min continuously awake) a FEES examination was indicated, to evaluate dysphagia severity, and to define the safest feeding route. FEES examination was conducted by two experienced SLPs according to a standardized FEES protocol^29^. In patients with a decreased level of consciousness, nutrition was applied via nasogastric tube (NGT).

In summary, the ability to swallow saliva, placebo pills, as well as different consistencies (honey-like thickened, liquid, semisolid, solid food) were evaluated, while documenting events of penetration and aspiration, and the extent of residues^30^. If a food consistency was swallowed, without penetration/aspiration and relevant residues, it was considered suitable/safe for oral intake.

Those patients passing the DA with no objective signs of dysphagia, but presented with facial palsy and/or loss of motor function of the bulbar muscle, were further tested with solid texture (1 slice of bread), to detect impaired bolus preparation^31^.

Patients, who did not receive a WST, due to severely impaired level of consciousness (reaction only to pain stimuli), were classified as secondary dysphagic^32^.

Recommendations regarding the oral diet, as well as the intake of the medication, was prescribed by the treating SLP.

### Assessment of feeding route

To assess the change in feeding route, the nutrition status prior and after SLP assessment were extracted from the medical file of each patient. Extent of oral restrictions were rated according to the Functional Oral Intake Scale (FOIS), with levels 1–3 indicating tube dependency, levels 4–6 total oral intake, and level 7 representing total oral intake with no restrictions^33^.

Furthermore, we evaluated any changes in administration of medication following SLP evaluation. The defined application routes were oral, parenteral, or enteral via feeding tube.

### Statistical analysis

The primary outcome variable for this analysis was the presence of a CIFR, as determined by an SLP and any defined by a change of the FOIS. Secondary outcome variables were predictors for tube dependency (FOIS 1–3) and a CIFR after SLP assessment.

Regarding baseline characteristics data are presented as medians (interquartile range, IQR) or means (depending on the presence of normal-distribution, tested by quantile-quantile plots), and numbers with percentages, unless otherwise indicated. The statistical significance of differences were assessed via Wilcoxon-matched-pair test, Mann-Whitney-U test for Friedmann test, depending on scale level and groups. Pearson’s square test or binomial test was used for binary variables, depending on scale level and groups. The SAPS II score was divided into thirds, using visual classification to assess the 33.3% patients with the highest mortality (SAPS II ≥ 38).

CIFR as the primary outcome variable was evaluated using binary logistic regression (unadjusted). Tube dependency, as a secondary outcome variable, was evaluated using adjusted logistic regression. Adjusted estimates of outcome (common odds ratio, odds ratio, and β) were calculated by taking the following variables into account: sex, age, length of stay ≥ 7 days, disease groups, pneumonia, kidney failure, intervention before ICU admission, ventilation, mRS ≥ 3, CCI, BMI, chronic lung disease, and maximal SAPS II score. The adjusted or unadjusted common odds ratios (OR) are reported with 95% confidence intervals (CI), which indicates statistical precision. The significance level was set to *p* < .05.

Statistical analysis was performed with SPSS version 26.0 (IBM) and GraphPad Prism 9.0 (GraphPad Software).

## Results

### Entire population

In total 497 patients were admitted to the Neuro-ICU for more than 48 h, between January 2018 to December 2018. Dysphagia assessment by an SLP was conducted in 148 patients, and absent in 349 cases, due to following reasons: (1) SLP was not consulted by the treating physician (*n* = 162, 32.6%), (2) patients were transferred ventilator-dependent to the rehabilitation hospital (*n* = 116, 23.3%) and (3) dysphagia assessment was not required due to palliative procedure (*n* = 71, 14.3%). Patients’ characteristics are displayed in Supplementary Table 1.

### Study cohort

We included 148 patients (70 female), with a mean age of 62.8 ± 17.29 years, 81 of which received neurological treatment. Details regarding the main diagnosis for ICU admission are displayed in the Table 1.

**Table 1:** Data is presented as number (%) or as median (25th to 75th percentiles, IQR) or mean ± standard deviation (SD). Intervention before ICU stay includes both, surgery and angiography.

	*n* = 148
Age (y; mean ± SD)	62.8 ± 17.29
Male	78 (52.7)
Length of stay (days)	11 (5–14)
Admission to Neurology/Neurosurgery	81/67
Maximal SAPS II (without GCS)	31.5 (26–40)
BMI [kg/m^2^]	27.0 ± 5.32
mRS before admission ≤ 2	123 (83.1)
Intervention before admission to ICU	69 (46.6)
**Disease categorisation**	
Ischemic stroke	39 (26.4)
Non-traumatic ICH	9 (6.1)
Non-traumatic SAH	29 (19.6)
Infectious encephalitis or meningitis	7 (4.7)
Epilepsy	8 (5.4)
Brain tumor	21 (14.2)
Traumatic brain injury	19 (12.8)
Other	16 (10.8)
**Comorbidities**	
Known neurological disease	75 (50.7)
Arterial Hypertension	80 (54.1)
Diabetes Mellitus	29 (19.6)
Cancer	33 (22.3)
Chronic lung disease	20 (13.5)
CCI	1 (0–3)
**Complications while ICU stay**	
Pneumonia	85 (57.4)
Any other infection	54 (36.5)
Kidney failure	13 (8.8)
Delirium	27 (18.2)
Death	9 (6.1)
**Mechanical ventilation**	109 (73.6)
Endotracheal Intubation	107 (72.3)
Intubation before admission	25 (16.9)
Reintubation	23 (15.5)
Tracheostomy	14 (9.5)
Ventilation hours	120.4 ± 180.96
Mechanical Ventilation ≥ 24 h	73 (49.3)
Mechanical Ventilation ≥ 7 days	40 (27.0)
Endotracheal tube size [mm]^+^	8 (7.5-8)
Max. cuff pressure [cmH_2_O]^+^	30 (30–30)

+: % of tube size and cuff pressure refers to total number of endotracheal ventilated patients (*n* = 107).

Body Mass Index, BMI; CCI, Charlson Comorbidity Index; Glasgow Coma Scale, GCS; ICH, intracranial haemorrhage; intensive care unit, ICU; modified Rankin Scale, mRS; SAPS II, Simplified-Acute-Physiology-Score II; SAH, subarachnoid haemorrhage.

Based on the DA, 125 (84.6%) patients were classified as dysphagic. For the sake of defining the safest feeding route, FEES could be completed in 63 subjects. Although indicated, 15 patients did not receive further instrumental swallowing diagnostics due to noncompliance (hyperactive delirium/agitation) or being transferred to a rehabilitation facility or another hospital, on short notice. The remaining 47 dysphagic patients were found to lack readiness for safe oral intake by the attending SLP, due to reduced vigilance (*n* = 41) or severe oral dysphagia (*n* = 6). Therefore, a FEES examination, for the purpose of defining the safest feeding route, was not performed. Either the SLP indicated the placement of an NGT or recommended to continue nutrition via the already existing feeding tube.

Based on dysphagia assessment by the SLP (DA/FEES), 98 patients were fully tube dependent (66.2%, FOIS = 1–3), while for 24 subjects total oral diet was indicated (4.7%, FOIS = 4; 7.4%, FOIS = 5; 4,1%, FOIS = 6). 26 patients were able to safely swallow without any dietary restrictions (17.6% FOIS = 7).

**Fig. 2:**
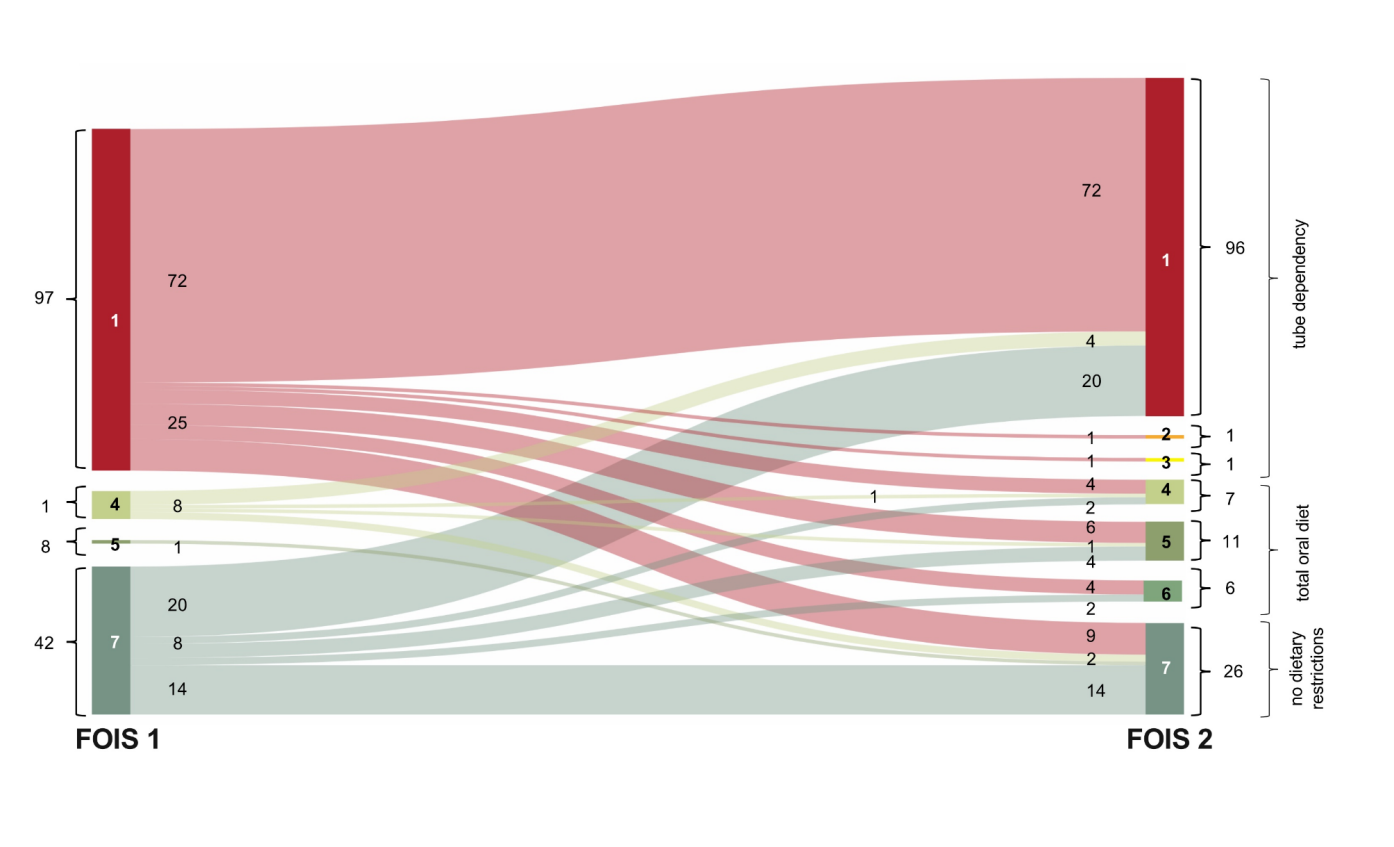
Sankey diagram. Functional Oral Intake Scale (FOIS) dysphagia assessments. A 7-point FOIS scale reflecting food and liquid intake by mouth on a consistent basis (7 being the best score: normal oral intake without any restrictions) was used before dysphagia assessment (DA) by a speech and language pathologist (FOIS 1) and after the DA by a speech and language pathologist (FOIS 2). Sankey diagram was created using SankeyMATIC by Steve Bogart (https://sankeymatic.com).

Dysphagia assessment by an SLP resulted in a significant CIFR in 61 (41.2%) patients, comparing FOIS 1 (prior CSE by SLP) to FOIS 2 (after CSE by SLP) (*p* < .001; OR 1.365, CI 1.195–1.558), represented in a Sankey diagram (Fig. 2).

Dietary restriction could be reduced in 29 (19.6%) cases, whereas 32 (21.6%) patients received stricter dietary recommendations, with 24 patients requiring NGT feeding withholding oral intake.

No CIFR following the SLP examination was observed in 87 (58.8%) patients. Hence, the SLP recommended maintaining enteral feeding via NGT, without any oral intake, in 72 cases (48.6%) due to FEES confirmed severe dysphagia or the lack of readiness for oral intake. In the remaining 15 patients the defined oral diet could be continued (*n* = 14, FOIS = 7; *n* = 1, FOIS = 4). Additionally, 69 patients (46.2%) experienced a change in medication delivery.

### Secondary outcomes

**Fig. 3:**
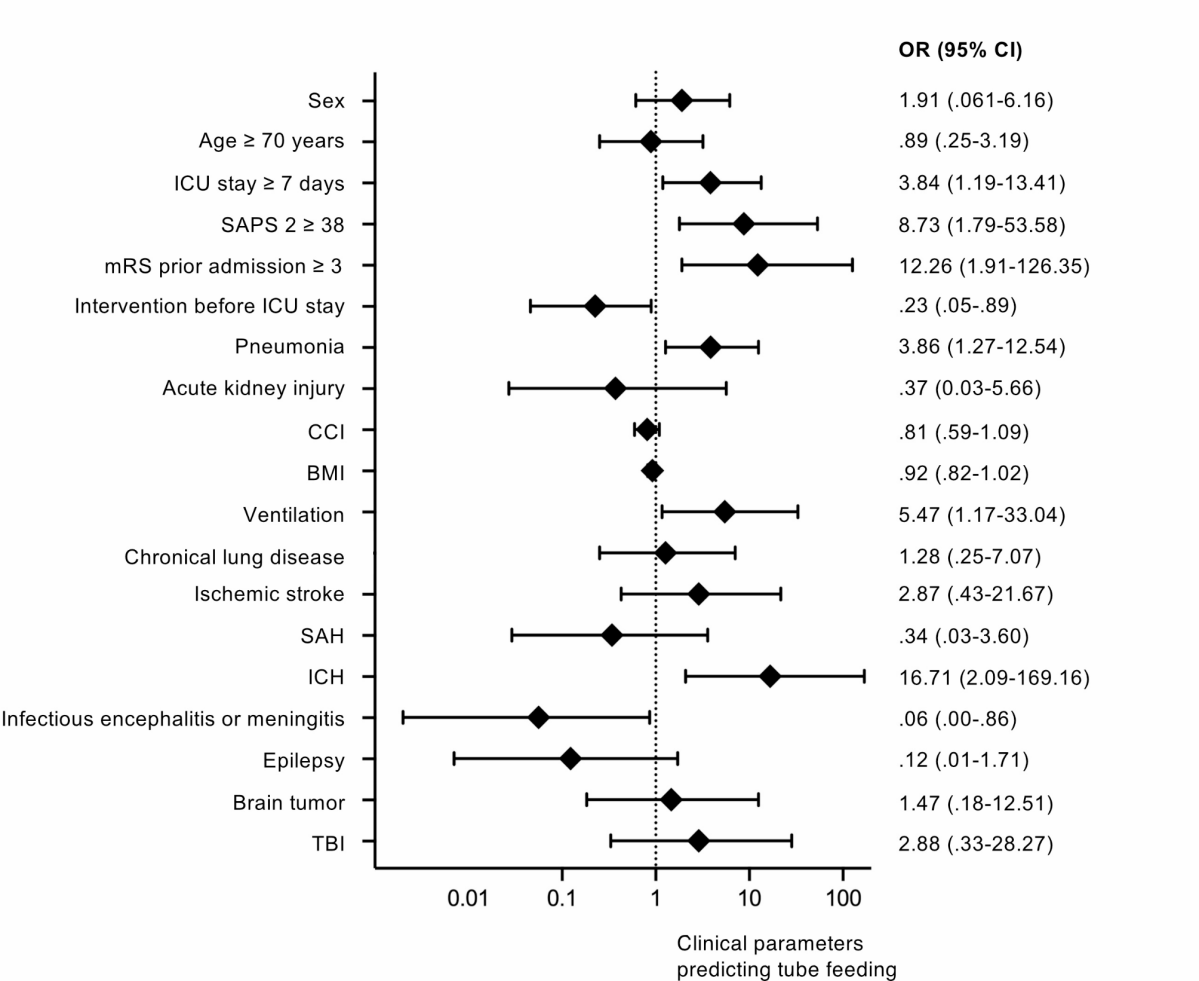
Forest plot showing association between nasogastric tube feeding and clinical variables of all patients with dysphagia assessment by a Speech and Language Pathologist. Body Mass Index, BMI; CCI, Charlson Comorbidity Index; ICH, intracranial hemorrhage; intensive care Unit, ICU; mRS, modified Rankin Scale; SAH, subarachnoid hemorrhage; SAPS II, Simplified-Acute-Physiology-Score II; TBI, traumatic brain injury.

Logistic regression analyses showed length of ICU stay ≥ 7 days (*p* = .028, OR 3.843, CI 1.187–13.408), mRS prior admission ≥ 3 (*p* = .018, OR 12.264, CI 1.906-126.343), mechanical ventilation (*p* = .043, OR 5.470, CI 1.165–33.040), SAPS II ≥ 38 (*p* = .012, OR 8.730, CI 1.794–53.579), pneumonia (*p* = .019, OR 3.857, CI 1.272–12.540) and intracerebral haemorrhage (*p* = .011, OR 16.707, CI 2.085-169.156), being associated with severe dysphagia necessitating tube feeding (Fig. 3).

Additionally, mechanical ventilation ≥ 24 h was significantly associated with an increased risk of tube dependency (χ²(1) = 11.281, *p* < .001), compared to those who were ventilated less than 24 h, or not ventilated at all. Dysphagic patients showed significantly longer duration of ventilation than non-dysphagic patients (z = 2.40, *p* = .017). As a conclusion a list of medical conditions/indicators to promote timely referral to an SLP for dysphagia assessment on the ICU is provided in Table 2.

Body Mass Index, BMI; CCI, Charlson Comorbidity Index; ICH, intracranial hemorrhage; intensive care Unit, ICU; mRS, modified Rankin Scale; SAH, subarachnoid hemorrhage; SAPS II, Simplified-Acute-Physiology-Score II; TBI, traumatic brain injury.

**Table 2:** Indicators for SLP consultation on neurological/neurosurgical ICU.

Admitted to ICU ≥ 7 days
Mechanical ventilation ≥ 24 h
Pre-mRS ≥ 3
SAPS II ≥ 38
Intracerebral haemorrhage
Pneumonia
Tube dependency
Altered level of consciousness

Intensive care Unit, ICU; mRS, modified Rankin Scale; SAPS II, Simplified-Acute-Physiology-Score II.

## Discussion

In this explorative retrospective study, we investigated to what extent dysphagia assessment by an SLP resulted in a change in the feeding route in Neuro-ICU patients. Furthermore, we tried to identify predictors for a CIFR, as well as for an increased dysphagia risk. In line with the existing literature, a high dysphagia frequency of about 85% was observed among our cohort. Following the dysphagia assessment by the SLP, about 60% of the dysphagic population required total tube feeding, while 20% of the cohort received an oral diet with food restrictions. Only in a minority of 20% no diet restrictions were necessary.

With regards to the defined feeding route, our data show a significant discrepancy between the assessment of the SLP, and the multiprofessional ICU team. Hence, a significant change of feeding route was observed after the dysphagia assessment by the SLP. In 20% of the patients, the SLP prescribed a stricter diet, whereby two-thirds of patients even required NGT feeding, withholding oral intake. Due to the comprehensive dysphagia assessment, including the broad use of FEES, aspiration, as well as relevant hypopharyngeal residues, could be detected. Consequently, dietary restriction and/or tube feeding were indicated, which may result in reduction of dysphagia associated complications, such as pneumonia or prolonged hospital stay^11^. Furthermore, early identification of dysphagia, and its underlying pathology, allows for a timely patient tailored intervention, reducing delayed return to oral intake, as well as pneumonia^34^.

On the other hand, SLP consultation resulted in reduction of dietary restrictions in approximately 20% of cases. Hence intake of multiple food textures was possible, and/or placed NGTs could be removed. Regarding the latter, there is clear evidence that the presence of NGT feeding is associated with colonization, and aspiration of pharyngeal secretions and gastric contents, leading to a high incidence of Gram-negative pneumonia^35^. Therefore, reduction of unnecessary NGT placement might also have a beneficial impact on the pneumonia rate.

The observed contrary opinion, between the SLP and the medical team, regarding the patients’ swallowing ability, might be attributed to different approaches in estimating swallowing function.

SLPs, commonly use structured methods to assess swallowing dysfunction, in awareness of symptoms, highly indicating dysphagia. Furthermore, in the present study the SLPs had easy access to FEES, which is known for its excellent sensitivity in detecting aspiration, and its accuracy in determining a safe diet^36^.

In contrast, nurses´ evaluation of swallowing ability is embedded in their clinical practice, on a continuing basis^10^. Additionally, interpretation of dysphagic symptoms might differ among the medical staff, and even within the same discipline. In this context, a focus group study, investigating perspectives on dysphagia management in ICU, showed that response to choking might result in a different conclusion, varying from NPO-status to continued oral feeding.

Furthermore, knowledge regarding e.g., what constitutes a safe swallow, or the limitations of clinical swallowing examination in estimating dysphagia or aspiration risk, differs among the professionals.

It often falls upon the nurses to assess the patients´ swallowing ability, to define the safest feeding route, when more specialized professionals are unavailable^10^. In this context, nurses relate lack of reaction during suctioning, or altered cognitive status, to the presence of dysphagia, while physicians focus on radiological findings (e.g., basal right pulmonary infiltrate), and medical history (repeated pulmonary infections)^9,10,15^.

Another interesting finding of our study was, that 50% of the patients, who were referred to the SLP for scrutinizing an oral diet, were found to lack readiness for safe oral intake. The main reason why the dysphagia professional considered these patients to be unfit for an oral diet was related to a reduced level of consciousness. Swallowing is a complex, dynamic sensorimotor process, involving voluntary and reflexive motor activity. In particular, the oral phase of swallowing is described as the voluntary (conscious) part of swallowing^32,37^. Therefore, certain components of swallowing e.g., lip prehension, lingual propulsion, or mastication might be severely affected by an altered state of consciousness, predisposing patients to an increased aspiration risk.

Therefore, the SLP refrained from bolus testing during DA, and/or from conducting FEES for the purposes of defining the safest diet, if vigilance or the oral phase was severely affected. Here, too, the judgement regarding the readiness of oral intake differed substantially between the critical care team and the dysphagia professional. This might be related to different approaches in estimating the swallowing ability, as well the lack of education, in terms of the necessary prerequisites for a safe oral intake.

Even if a patient is in a medically stable condition, responding and/or following commands, and showing eye-opening, the oral phase might be ineffective, as it requires a certain level of awareness and arousal. A more profound investigation of swallowing, including examination of the bulbar muscles, cranial nerves, or reflexes (e.g., gag and swallowing reflex) etc., could unmask dysphagic symptoms, which remain unrecognized, if not evaluated.

Additionally, our investigation showed that about 30% of the ICU patients were not referred to the SLP for dysphagia assessment by the treating physician, due to unknown reasons. As no implemented standard operating procedures for dysphagia exist yet, it is left to the discretion of the physician and/or the nurse in charge, to decide if a DA by an SLP is necessary.

Hence, the critical care team assessed dysphagia-related risk on an individual basis^38,39^. However, the results of our study show that the presence of dysphagia and/or lack of readiness of oral intake might be misjudged by the ICU-team, and thus expose patients to a higher risk for pneumonia and malnutrition.

Besides a standardized algorithm for estimating ICU-dysphagia, knowledge of clinical predictors for dysphagia might enhance timely referral to an SLP. In this context, we found length of ICU ≥ 7 days, undergoing mechanical ventilation, pneumonia, pre-mRS ≥ 3, SAPS II ≥ 38 and intracerebral haemorrhage, being significantly associated with severe dysphagia, is in line with previous studies. Also, prolonged mechanical ventilation was strongly related to an increased risk of tube dependency.

In summary, our findings are in line with others, reporting on a significant association between the presence of ICU-dysphagia, and the clinical status of the patients before ICU-admission. Pre-existing disabilities have an impact on the functional reserve of the swallowing system´s adjustment to an additive condition. Furthermore, although, if not novel, our data also show a strong correlation between mechanical ventilation and altered swallowing function. Endotracheal trauma, dyssynchronization of breathing and swallowing, and decreased pharyngeal sensation, are discussed as contributing factors.

To summarize, dysphagia is markedly present in patients in the Neuro-ICU. Although, the critical care team is highly trained and specialized, our data suggest a knowledge gap in identifying patient with or at risk of dysphagia, which may result in an incorrect determination of the appropriate feeding route.

In contrast to acute stroke management, in which patients with suspected dysphagia remain nil per os until dysphagia screening by an SLP is completed, standardized swallowing evaluation, as well as frequent application of instrumental swallowing diagnostics, to define the safest feeding route, have not yet been widely implemented (34). Considering the high prevalence of dysphagia in ICUs, and its devastating impact on patients’ outcome, comprehensive dysphagia assessment by a trained SLP is definitely warranted.

Limitations of our study are its retrospective design, and the small sample size, limiting a dedicated analysis of predictors for a change in feeding route. Furthermore, due to the retrospective design, and a lack of a control group, we cannot comment on the impact of the patient’s outcome, following the dysphagia assessment by the SLP. With regards to our patient selection, our findings cannot be applied to non-neurological ICUs.

## Conclusion

Dysphagia is a frequent finding in the Neuro-ICU. A comprehensive dysphagia assessment by an SLP is associated with a significant change in feeding. Furthermore, the assessment regarding the readiness of oral intake, differs significantly between the dysphagia professional and the critical care team.

Although, our study showed a significant discrepancy in recommendations for the feeding route between the SLP and the ICU-team, it remains unclear if a standardized DA by a dysphagia expert -when to compared to the assessment of the multidisciplinary ICU-team- leads to an improvement in patient related outcomes (e.g., reduction of pneumonia rate, earlier discharge from ICU or reduction of extubation failure). The implementation of a dysphagia screening protocol before first oral intake on stroke units resulted in reduction of pneumonia and mortality rates. We strongly recommend that further research should be conducted to determine whether such procedures might have the same impact in the ICU.

Our data emphasizes the need for a standardized operating procedure, for estimating dysphagia risk, and the readiness for oral intake. Furthermore, an ICU-specific trained SLP, as well as timely availability of instrumental swallowing diagnostics (e.g., FEES), should be mandatory for the Neuro-ICUs, if not all ICUs.

## Electronic supplementary material

Below is the link to the electronic supplementary material.

Supplementary Material 1

## Data Availability

The datasets generated and analysed during the current study are not publicly available due to restrictions e.g. their containing information that could compromise the privacy of research participants but are available from the corresponding author on reasonable request.

## Abbreviations

BMI: Body mass index
CCI: Charlson comorbidity index
CI: Confidence interval
CIFR: Change in feeding route
CIMA: Change in medication application
DA: Dysphagia assessment
FEES: Fiberoptic endoscopic evaluation of swallowing
FOIS: Functional Oral Intake Scale
ICU: Intensive care unit
IQR: Interquartile range
mRS: Modified Rankin Scale
NGT: Nasogastric tube
PED: Post extubation dysphagia
SAPSII: Simplified Acute Physiology Score II
SD: Standard deviation
SLP: Speech and language pathologist
WST: Water swallowing test

## Declarations

### Guidelines

The manuscript was prepared according to STROBE reporting guidelines.
