# Supplementary material for: Intensive care unit-acquired dysphagia – change in feeding route after a standardized dysphagia assessment in neurocritical care patients
Source: Sci Rep. 2024 Dec 2;14:29993. doi: 10.1038/s41598-024-81529-1 (PMC11612384; doi:10.1038/s41598-024-81529-1)
Supplement: Supplementary file 1 — Supplementary Material 1 [file 41598_2024_81529_MOESM1_ESM.docx]

**Supplementary Materials**

|  | **SLP assessment** | **No SLP assessment** |  |
| --- | --- | --- | --- |
|  | **n=148** | **n=349** | **p-value** |
| Age (y; mean ± SD) | 62.8 ± 17.29 | 64.0 ± 17.01 | .475 |
| Male | 78 (52.7) | 205 (58.7) | .178 |
|  |  |  |  |
| Neurological patient | 81 (57.7) | 108 (30.9) | <.001 |
| Neurosurgical patient | 67 (45.3) | 241 (60.1 |  |
|  |  |  |  |
| **Diagnosis** |  |  |  |
| Ischemic stroke | 39 (26.4) | 38 (10.9) | .003 |
| Non-traumatic ICH | 9 (6.1) | 50 (14.3) | .009 |
| Non-traumatic SAH | 29 (19.6) | 49 (14.0) | .120 |
| Infectious encephalitis or meningitis | 7 (4.7) | 9 (2.6) | .214 |
| Epilepsy | 8 (5.4) | 13 (3.7) | .349 |
| Brain tumor | 21 (14.2) | 42 (12.0) | .509 |
| Traumatic brain injury | 19 (12.8) | 85 (24.4) | .004 |
| Others | 16 (10.8) | 63 (18.1) | .008 |

**Supplementary Table 1:** Data is presented as number (%) or mean ± standard deviation (SD).

SLP, Speech and Language Pathologist; ICH, intracranial haemorrhage; ICU, intensive care unit; SAH, subarachnoid haemorrhage; SD, standard deviation.
